# Elevated mRNA expression and defective processing of cathepsin D in HeLa cells lacking the mannose 6‐phosphate pathway

**DOI:** 10.1002/2211-5463.13169

**Published:** 2021-05-05

**Authors:** Lin Liu, Balraj Doray

**Affiliations:** ^1^ Department of Internal Medicine Washington University School of Medicine St. Louis MO USA; ^2^ Present address: M6P Therapeutics 20 S. Sarah Street St. Louis MO 63108 USA

**Keywords:** cathepsin B, cathepsin D, cathepsin L, GlcNAc‐1‐phosphotransferase, lysosomes, mannose 6‐phosphate pathway

## Abstract

Disruption of the mannose 6‐phosphate (M‐6‐P) pathway in HeLa cells by inactivation of the *GNPTAB* gene, which encodes the α/β subunits of GlcNAc‐1‐phosphotransferase, results in missorting of newly synthesized lysosomal acid hydrolases to the cell culture media instead of transport to the endolysosomal system. We previously demonstrated that the majority of the lysosomal aspartyl protease, cathepsin D, is secreted in these *GNPTAB* ^−/−^ HeLa cells. However, the intracellular content of cathepsin D in these cells was still greater than that of WT HeLa cells which retained most of the protease, indicating a marked elevation of cathepsin D expression in response to abrogation of the M‐6‐P pathway. Here, we demonstrate that HeLa cells lacking GlcNAc‐1‐phosphotransferase show a fivefold increase in cathepsin D mRNA expression over control cells, accounting for the increase in cathepsin D at the protein level. Further, we show that this increase at the mRNA level occurs independent of the transcription factors TFEB and TFE3. The intracellular cathepsin D can still be trafficked to lysosomes in the absence of the M‐6‐P pathway, but fails to undergo proteolytic processing into the fully mature heavy and light chains. Uptake experiments performed by feeding *GNPTAB* ^−/−^ HeLa cells with various phosphorylated cathepsins reveal that only cathepsin B is capable of partially restoring cleavage, providing evidence for a role for cathepsin B in the proteolytic processing of cathepsin D.

## Graphical abstract

Cathepsin D mRNA expression is increased fivefold in HeLa cells when the Man‐6‐P pathway is inactivated. This increase is independent of both TFEB and TFE3. These cells also lack cathepsin B, which is partly responsible for the failure of cathepsin D processing to occur. The effect is reversed by feeding the knockout HeLa cells with cathepsin B.

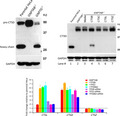

**Abbreviations**
GlcNAc‐1‐phosphotransferase: UDP‐GlcNAc:lysosomal enzyme N‐acetylglucosamine‐1‐phosphotransferase
M‐6‐P: mannose 6‐phosphate
TFEB: Transcription Factor EB
TFE3: Transcription Factor E3

The proper delivery of the 60 or so newly synthesized acid hydrolases to the lysosome of a cell is essential for the degradation of intracellular and endocytosed materials within this organelle [1]. Key among these enzymes are the lysosomal proteases known as cathepsins, all of which are initially made as inactive pre‐proenzymes in the endoplasmic reticulum (ER) [2]. Following removal of the signal peptide within the lumen of the ER, the proenzyme, also called a zymogen, acquires N‐linked glycans as it transits from the ER through the compartments of the Golgi. Transport of the pro‐cathepsins from the trans‐Golgi network (TGN) to the endolysosomal system via the mannose 6‐phosphate (M‐6‐P)‐dependent pathway requires tagging of these acid hydrolases with the M‐6‐P moiety on their high‐mannose glycans in the *cis*‐Golgi, a process mediated by the enzyme UDP‐GlcNAc:lysosomal enzyme N‐acetylglucosamine‐1‐phosphotransferase (GlcNAc‐1‐phosphotransferase) [3]. Upon reaching the lysosome, the pro‐cathepsins undergo specific proteolytic cleavage events for catalytic activation.

Cathepsin D is the most ubiquitously expressed lysosomal aspartyl protease with especially high levels in the human brain [4]. In addition to the metabolic breakdown of proteins in the lysosome, cathepsin D is involved in a diverse array of physiologic processes, including activation of growth factors, hormones and enzyme precursors, processing of antigens, and regulation of apoptosis [5]. Mutations of the human gene encoding cathepsin D, *CTSD*, that severely impair enzyme activity gives rise to the lysosomal storage disorder neuronal ceroid lipofuscinosis [6]. Cathepsin D has also been implicated in a number of other pathological conditions including atherosclerosis, cancer, cardiovascular disease, and neurodegenerative disorders such as Alzheimer’s, Huntington’s, and Parkinson’s diseases [7].

During the course of studying a HeLa cell line (*GNPTAB* ^−/−^ cells) with inactivation of the M‐6‐P pathway, we found that although 80% of the newly synthesized cathepsin D was secreted by these cells, the remaining 20% of intracellular cathepsin D exceeded the level observed with the parental HeLa cells, wherein upward of 90% of the newly synthesized cathepsin D was retained [8]. This indicated that cathepsin D protein expression was markedly elevated in the *GNPTAB* ^−/−^ HeLa cells. Moreover, we and others noted that these cells accumulate only pro‐cathepsin D while failing to convert this single‐chain molecule to the two‐chain form [8, 9, 10]. Here, we demonstrate that in the absence of the M‐6‐P pathway, cathepsin D gene expression in HeLa cells is elevated fivefold at the mRNA level but this upregulation occurs independent of either Transcription Factor EB (TFEB) or Transcription Factor E3 (TFE3), two transcription factors that function as master regulators of lysosomal homeostasis [11]. We further determine that it is the absence of cathepsin B that is responsible for the failure to detect cathepsin D processing in the *GNPTAB* ^−/−^ HeLa cells.

## Materials and methods

### Cell lines

Generation of the *GNPTAB* ^−/−^ and *GNPTG* ^−/−^ HeLa cell line has been described in detail previously [12]. Mutant and parental HeLa cell lines were maintained in Dulbecco’s Modified Eagle Medium (Life Technologies, Carlsbad, CA, USA), while Expi293 cells (Life Technologies) were grown in suspension in Expi293 expression medium (Life Technologies). All media contained 0.11 g·L^−1^ sodium pyruvate and 4.5 g·L^−1^ glucose, supplemented with 10% (vol/vol) FBS (Atlanta Biologicals, Flowery Branch, GA, USA), 100 000 U·L^−1^ penicillin, 100 mg·L^−1^ streptomycin (Life Technologies), and 2 mM l‐glutamine (Life Technologies).

### Antibodies

The following antibodies were used in this study: anti‐V5 mouse monoclonal antibody (Thermo Fisher Scientific, Waltham, MA, USA Cat #R960‐25), anti‐cathepsin D rabbit polyclonal antibody (generated in our lab), anti‐Lamp1 mouse monoclonal antibody (H4A3) (Development Studies Hybridoma Bank, Iowa City, IA, USA), anti‐cathepsin B 3E4 mouse monoclonal antibody (a generous gift from Bonnie Sloane, Martin Luther University, Halle‐Wittenberg, Germany), and anti‐cathepsin L goat polyclonal antibody (R&D Systems, Inc. Minneapolis, MN, USA Cat #AF1515).

### DNA constructs

The construction of the full‐length *GNPTAB* cDNA [12] and the truncated S1‐S3 cDNA [13], both in the vector pcDNA6/V5‐His, has been described. The cDNAs for expression of cathepsins B, C, L, and Z in Expi 293 cells were all purchased from OriGene (Rockville, MD, USA).

### Immunofluorescence microscopy

*GNPTAB* ^−/−^, *GNPTG* ^−/−^, and parental Hela cells were fixed with 4% formaldehyde (Sigma‐Aldrich, St. Louis, MO, USA) and permeabilized in 0.1% (vol/vol) Triton X‐100 in PBS. Cells were blocked for 1 h with 2% IgG‐free BSA (Jackson Immuno‐Research, West Grove, PA, USA) and probed with the indicated combinations of antibodies as described in the figure legend. The images were acquired with an LSM880 confocal microscope (Carl Zeiss, Inc., Peabody, MA, USA) in the Molecular Microbiology Imaging Facility at Washington University School of Medicine in St. Louis. Images were analyzed by imagej software (NIH, Bathesda, MD, USA).

### Reverse transcriptase quantitative PCR (RT‐qPCR)

Total RNA was extracted with TRIzol reagent (Thermo Fisher Scientific) from parental HeLa, *GNPTAB* ^−/−^, and *GNPTG* ^−/−^ cells according to the manufacturer’s instructions. RNA concentration was determined by measuring the OD_260_ value. Two hundred nanogram of RNA from each cell line was first reverse‐transcribed using Omniscript RT Kit (Qiagen, Hilden, Germany) in a total volume of 20 µL to produce first‐strand cDNA. qPCR was then carried out using a StepOnePlus thermocycler (Applied Biosystems, Foster City, CA, USA). The reaction was performed in triplicate using SYBR Green Master Mix (Bio‐Rad, Hercules, CA, USA) following the manufacturer’s protocol, and gene expression was calculated by either the 2^‐ΔCT^ or the 2^–∆∆CT^ method, using the human *ACTB* gene as the reference gene.

The following primers were used in this study for RT‐qPCR:

*CTSB* fwd ^5′^GAATGGCACACCCTACTGG^3′^

rev ^5′^TGATCGGTGCGTGGAATTC^3′^

*CTSC* fwd ^5′^AACTGCACCTATCTTGACCTG^3′^

rev ^5′^CTGTATCCAGCTTCTGAAGGTAC^3′^

*CTSD* fwd ^5′^GGACTACACGCTCAAGGTG^3′^

rev ^5′^GTTGTCACGGTCAAACACAG^3′^

*CTSF* fwd ^5′^CCCTTGGCTCATTGACCATG^3′^

rev ^5′^GGACCCACGATGCAAGTAG^3′^

*CTSH* fwd ^5′^AACTGTGCGTGAACTCCTTAG^3′^

rev ^5′^TGTGGGCGTTTATCTTCCTC^3′^

*CTSK* fwd ^5′^GAAGACCCACAGGAAGCAATA^3′^

rev ^5′^TGTATGGACACCAAGAGAAGC^3′^

*CTSL* fwd ^5′^TTTGAGCCAGACTGTAGCAG^3′^

rev ^5′^ATCTTTACGTAGCCACCCATG^3′^

*CTSO* fwd ^5′^GTGGAGAAGCAAATCATGCAG^3′^

rev ^5′^GCAATACCACAAACATTACTTCCC^3′^

*CTSS* fwd ^5′^TGTGGTTGGCTATGGTGATC^3′^

rev ^5′^TTTCTGGGTAAGAGGGAAAGC^3′^

*CTSV* fwd ^5′^TGGATCATGGTGTTCTGGTG^3′^

rev ^5′^CAGTGGTTGTTCTTGTCTTTGG^3′^

*CTSW* fwd ^5′^AGTACCTTTCAGCTGTGACTG^3′^

rev ^5′^CGTCCACAAAATCCCAGAAAC^3′^

*CTSZ* fwd ^5′^TGGATGGTGTCAACTATGCC^3′^

rev ^5′^GCTCCCTTCCTCTTGATGTTG^3′^

*ACTB* fwd ^5′^CCCAGCACAATGAAGATCAAG^3′^

rev ^5′^GACTCGTCATACTCCTGCTTG^3′^

*TFEB* fwd ^5′^CTGACCCAGAAGCGAGAG^3′^

rev ^5′^TCAGCATTCCCAACTCCTTG^3′^

*TFE3* fwd ^5′^CCTGCAGCTCCGAATTCAG^3′^

rev ^5′^CTGTCAGAAGCCGAAGTCG^3′^

### Enzyme expression in Expi293 cells and enzyme uptake assays

Expi293 cells in suspension were cotransfected with the cDNAs of cathepsins B, C, D, L, and Z, along with the *GNPTAB* S1‐S3 mutant construct, as described [13]. Media was collected aseptically 2–3 days post‐transfection for use in uptake assays. For cell uptake experiments, *GNPTAB* ^−/−^ HeLa cells were plated on a 12‐well plate at ~ 80% confluence 1 day prior to the uptake experiment. The total protein concentration of the media containing the various secreted cathepsins was measured using the Bradford Assay (Bio‐Rad) and determined to be roughly equivalent. Fifty microlitre aliquots of this media containing each enzyme from the producing cells were then added to the *GNPTAB* ^−/−^ HeLa cells in a final volume of 500 μL. The cells were incubated with the media for 24 h before being collected and processed for immunoblotting.

### Immunoblotting

Proteins resolved by using SDS/PAGE under reducing conditions were transferred to nitrocellulose membrane and detected with antibodies as described in the figure legends. Equal amounts of whole‐cell extract were loaded on the gels. For experiments with E‐64 and pepstatin A, parental HeLa or *GNPTAB* ^−/−^ HeLa cells were transfected with the human *GNPTAB*‐V5/His cDNA in pcDNA6 using Lipofectamine 3000 (Thermo Fisher Scientific) according to the manufacturer’s protocol. Cells were treated with the indicated concentrations of the protease inhibitors for 24 h. Control cells were treated with only vehicle DMSO.

## Results

### *CTSD* gene expression is highly elevated in *GNPTAB* ^−/−^ HeLa cells with partial retention of the synthesized cathepsin D

**Fig. 1:**
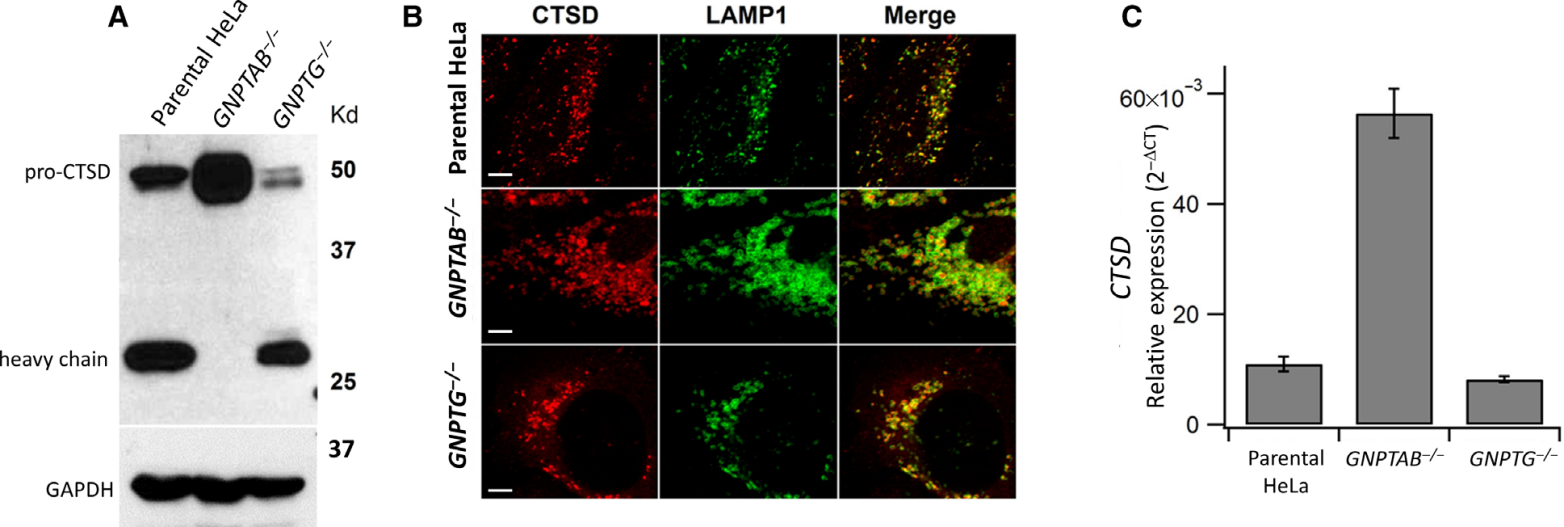
Analysis of cathepsin D in WT, *GNPTAB* ^−/−^, and *GNPTG* ^−/−^ HeLa cells. (A) Immunoblot of endogenous cathepsin D expression in whole‐cell lysates of the indicated cell lines, as determined by probing with an anti‐cathepsin D antibody. (B) Confocal immunofluorescence images of WT, *GNPTAB* ^−/−^, and *GNPTG* ^−/−^ HeLa cells, stained for cathepsin D and colocalized with the late endosomal/lysosomal marker LAMP1. Scale bars correspond to 5 μm. (C) Total RNA was isolated from the three cell lines, and cathepsin D mRNA levels were measured by RT‐qPCR using the *ACTB* gene (encoding β‐actin) as the reference gene for normalization, as described under Materials and Methods. Error bars represent the mean ± SEM of three individual determinations for the various cell lines. *P* = 0.00007 (Student’s t‐test) for change in *CTSD* expression in *GNPTAB* ^−/−^ cells relative parental HeLa.

The *GNPTAB* ^−/−^ HeLa cell line lacks the α/β subunits GlcNAc‐1‐phosphotransferase, the enzyme that generates the M‐6‐P moiety on lysosomal enzymes [3]. Consequently, the bulk of the acid hydrolases synthesized by these cells are secreted rather than sorted to lysosomes [12]. Thus, it was surprising when our laboratory determined that the cathepsin D content of these cells was actually higher than either WT parental or *GNPTG* ^−/−^ HeLa cells [8] (Fig. 1A). The *GNPTG* gene encodes the γ subunit of GlcNAc‐1‐phosphotransferase, which enhances the phosphorylation of a subset of acid hydrolases, but is dispensable for the catalytic activity of the enzyme [12, 14].

In a recent study using pulse‐chase experiments, we found that 93% of the newly synthesized cathepsin D of WT HeLa cells remained intracellular within a 5‐h time period. This is in stark contrast to the *GNPTAB* ^−/−^ HeLa cells where only 22.5% of the newly synthesized cathepsin D remained intracellular, with 77.5% being secreted into the cell culture medium within the same time period [8]. Still, the amount of the partially retained cathepsin D within the latter cells exceeded the level seen in the WT cells, indicating that in the absence of the M‐6‐P pathway, HeLa cells either upregulate expression of cathepsin D and/or decrease the turnover rate of the protein within lysosomes. Moreover, only pro‐cathepsin D was detected in the immunoblot of whole‐cell lysates from *GNPTAB* ^−/−^ cells, whereas the fully processed heavy chain, in addition to the proform, was seen with both WT and *GNPTG* ^−/−^ HeLa cells [8] (Fig. 1A). A similar observation has been reported by Boonen *et al*. [9] and Miller *et al*. [10] with our *GNPTAB* ^−/−^ cells. Despite the absence of GlcNAc‐1‐phosphotransferase, confocal immunofluorescence microscopy showed that the pro‐cathepsin D in the *GNPTAB* ^−/−^ cells colocalized with the lysosomal membrane protein, LAMP1, in enlarged late endosomes/lysosomes, indicating transport of the aspartyl protease to this organelle (Fig. 1B). The morphology of the late endosomes/lysosomes of the *GNPTG* ^−/−^ cells was intermediate between WT and *GNPTAB* ^−/−^ cells, as previously reported [12].

Reverse transcriptase quantitative PCR (RT‐qPCR) was next performed to assess the level of cathepsin D mRNA in the three cell lines. Using total RNA isolated from the individual cell lines, RT‐qPCR analysis determined that the cathepsin D mRNA level was markedly elevated (between fivefold to sixfold) in the *GNPTAB* ^−/−^ cells compared with the WT and *GNPTG* ^−/−^ cells (Fig. 1C).

### Elevation of cathepsin D and cathepsin L gene expression in *GNPTAB* ^−/−^ HeLa cells occurs independent of TFEB and TFE3

**Fig. 2:**
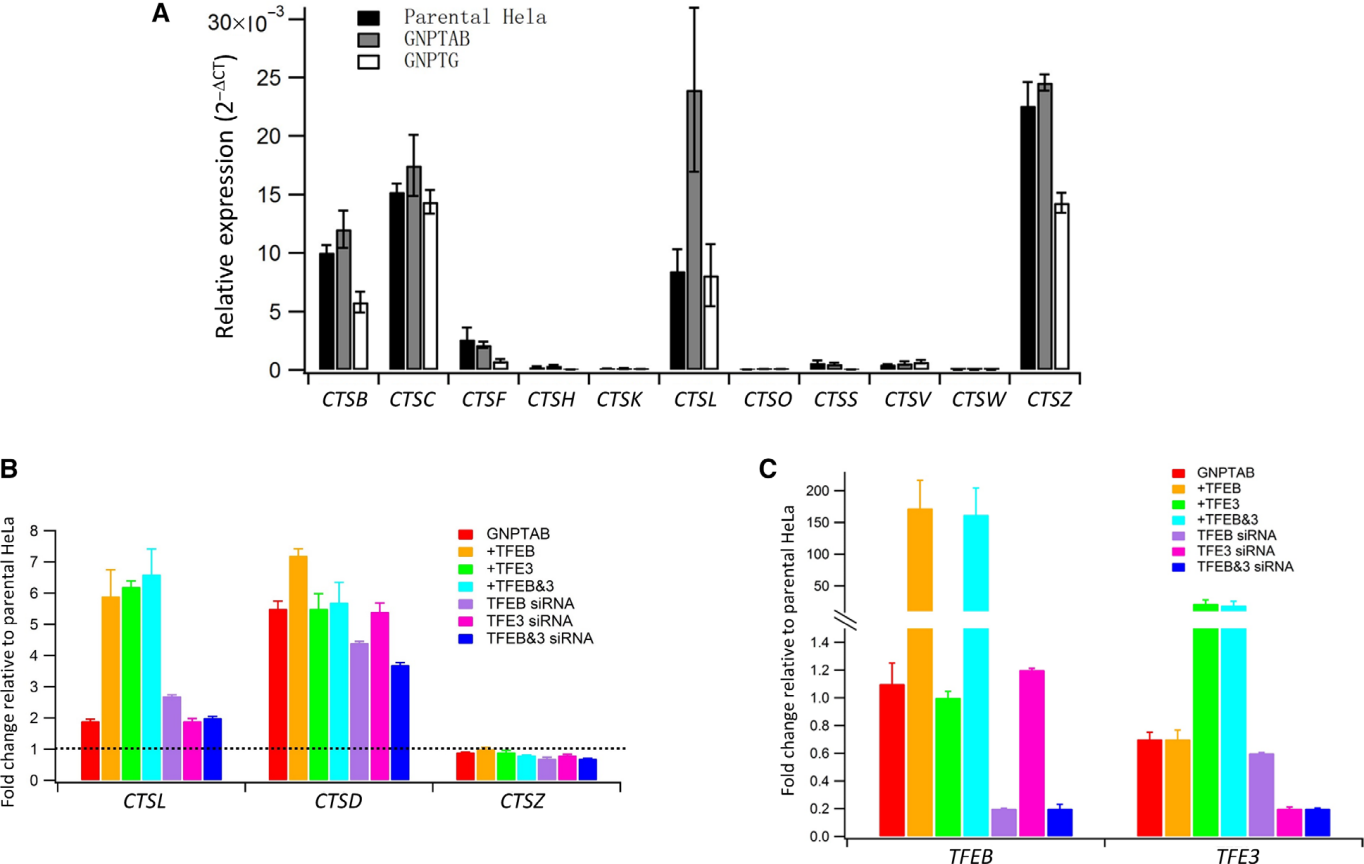
Analysis of the expression of *CTS* genes in WT, *GNPTAB* ^−/−^, and *GNPTG* ^−/−^ HeLa cells. (A) Total RNA was isolated from the three cell lines, and the mRNA levels of the indicated cathepsins were measured by RT‐qPCR using the *ACTB* gene as the reference gene for normalization. Delta Ct was calculated using the *ACTB* qPCR Ct value minus the qPCR Ct values of the various cathepsins. Error bars represent the mean ± SEM of three individual determinations for the various cell lines. The following *P* values (Student’s t‐test) were obtained for the change in gene expression between *GNPTAB* ^−/−^ cells and parental HeLa; *CTSB P* = 0.11286, *CTSC P* = 0.21899, *CTSL P* = 0.02059, and *CTSZ P* = 0.18135. (B) Total RNA was isolated from either untransfected *GNPTAB* ^−/−^ cells (red), or cells transfected with plasmids expressing TFEB and TFE3, individually or in combination, or cells transfected with oligonucleotides to silence TFEB and TFE3, individually or in combination. The level of the indicated cathepsins under the various conditions was then measured by RT‐qPCR using the *ACTB* gene as the reference gene for normalization. (C) The same cells used in (B) were also analyzed for the message levels of either TFEB or TFE3 using the *ACTB* gene as the reference gene for normalization. Error bars represent the mean ± SEM of three individual determinations for the different conditions (B and C).

Since the mRNA level of cathepsin D was markedly elevated when the *GNPTAB* gene was inactivated in HeLa cells, we next asked if expression of other cathepsins at the mRNA level was also altered in the *GNPTAB* ^−/−^ cells. Of the 11 cathepsins tested, RT‐qPCR analysis showed that only five had detectable mRNA levels in WT HeLa cells (*CTSB*, *CTSC*, *CTSF*, *CTSL,* and *CTSZ*), and of these, only the mRNA of *CTSL* was also increased in the *GNPTAB* ^−/−^ cells, relative to either WT or *GNPTG* ^−/−^ cells (Fig. 2A). The slightly higher mRNA levels of *CTSB*, *CTSC*, and *CTSZ* under these conditions were not statistically significant.

Cathepsin D gene expression has been shown to be regulated by TFEB, a member of the MiT/TFE family, which binds to a common E‐box response element termed CLEAR (Coordinated Lysosomal Expression and Regulation), found in the promoter region of many lysosomal genes [15]. In addition, TFE3, a closely related paralog of TFEB, was also shown to upregulate expression of cathepsin D, and it was proposed that TFE3 controls lysosomal biogenesis by regulating an overlapping gene network [16]. We, therefore, asked if either TFEB or TFE3, or both could be responsible for the highly increased gene expression of *CTSD* observed in *GNPTAB* ^−/−^ HeLa cells. Total RNA isolated from these cells was subject to RT‐qPCR to assess the mRNA levels of cathepsins D, L, and Z under basal, TFEB/TFE3 overexpression, or TFEB/TFE3 siRNA conditions. Under basal conditions, *CTSL* gene expression was increased twofold in *GNPTAB* ^−/−^ HeLa cells relative to WT parental HeLa cells, which is set at 1 (Fig. 2A and 2B, CTSL). The mRNA level of the *CTSL* gene was increased a further threefold upon overexpression of TFEB and TFE3 in *GNPTAB* ^−/−^ cells, either individually or in combination, relative to the untransfected *GNPTAB* ^−/−^ cells (Fig. 2B, CTSL). In contrast to *CTSL* (twofold increase in gene expression under basal conditions), *CTSD* gene expression was elevated fivefold to sixfold in *GNPTAB* ^−/−^ cells relative to WT cells (Figs 1C and 2B, CTSD). However, any further increase in *CTSD* mRNA expression when TFEB was overexpressed was only marginal (Fig. 2B, CTSD). The overexpression of TFE3, in this case, did not elicit an increase, as was the case when TFEB and TFE3 were coexpressed together. It should be noted that the mRNA level of TFEB is not significantly different between the parental WT and *GNPTAB* ^−/−^ HeLa cells (Fig. 2C, TFEB), while the mRNA level of TFE3 is ~ 30% lower in *GNPTAB* ^−/−^ HeLa cells relative to WT cells, which is set at 1 (Fig. 2C, TFE3).

We next performed RNA interference (RNA‐i) of TFEB and TFE3. Knocking down TFEB or TFE3, either alone or in combination, did not significantly decrease *CTSD* or *CTSL* gene expression in *GNPTAB* ^−/−^ HeLa cells (Fig. 2B, CTSL and CTSD). The expression of *CTSZ* remained unchanged under any circumstances in these experiments (Fig. 2B, CTSZ). This result was somewhat surprising, especially when TFEB and TFE3 were overexpressed, since the promoter region of the *CTSZ* gene has been shown to contain a CLEAR element [15]. However, it is worth noting that the *CTSZ* gene was not identified among the list of lysosomal hydrolase genes that represent the most likely direct targets of TFEB in HeLa cells [17].

The specific effects of either overexpression or RNA‐i of TFEB and TFE3, on their respective mRNA levels, were confirmed by the RT‐qPCR data shown in Fig. 2C. Taken together, these data indicate that the markedly increased expression of the *CTSD* gene in *GNPTAB* ^−/−^ cells is likely occurring through a mechanism that is independent of TFEB and TFE3.

### The retained cathepsin D in *GNPTAB* ^−/−^ cells does not undergo maturation to the two‐chain species

**Fig. 3:**
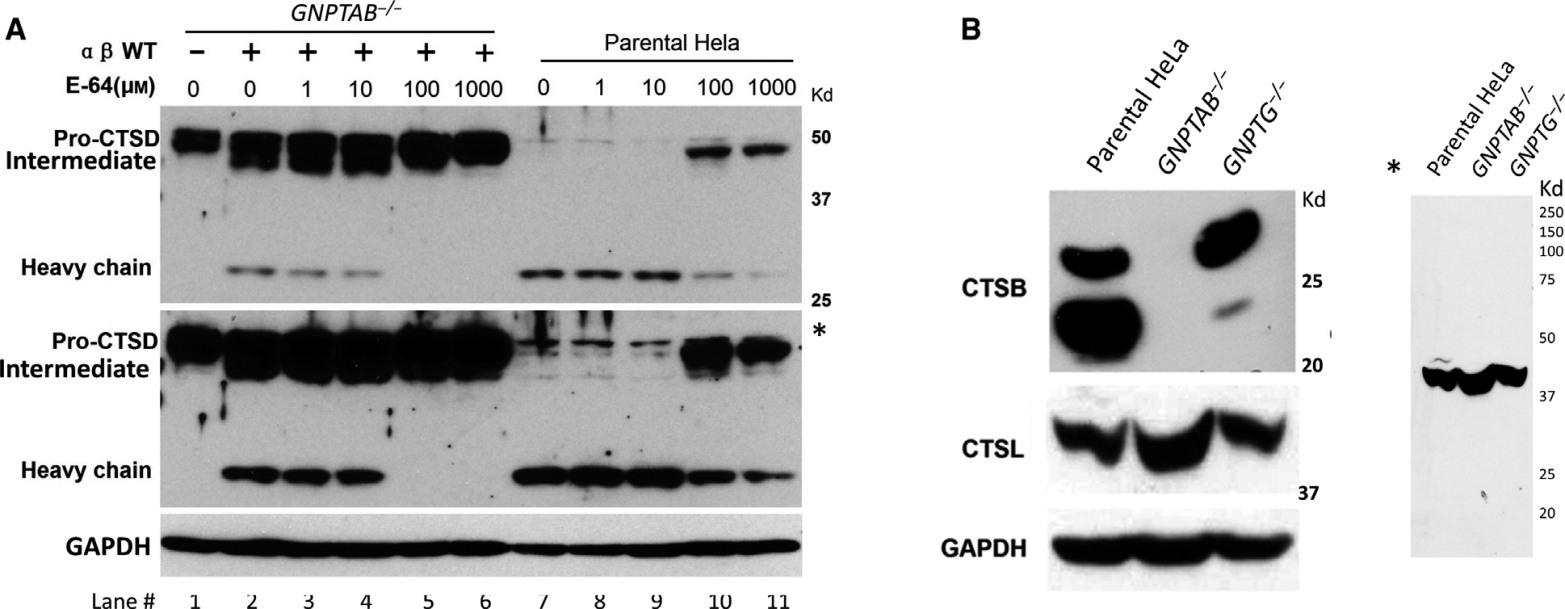
Effect of the cysteine protease inhibitor E‐64 on cathepsin D processing. (A) *GNPTAB* ^−/−^ cells transfected with the human *GNPTAB‐*V5/His cDNA construct, and untransfected WT parental HeLa cells were treated with the indicated concentrations of E‐64 for 24 h. Equivalent amount of whole‐cell lysates were loaded for immunoblotting and probed for endogenous cathepsin D. * Indicates a longer exposure of the cathepsin D blot. (B) Immunoblot of endogenous cathepsin B and cathepsin L in whole‐cell lysates of the indicated cell lines, as determined by probing with their respective antibodies. * Indicates a longer exposure of the whole cathepsin L blot.

While the level of the retained cathepsin D in the *GNPTAB* ^−/−^ cells exceeded that of the WT cells, there was a complete absence of the 30 kDa form, indicating that the proform of the protease was not being converted to the mature two‐chain form of the enzyme. Transfection of these cells with the α/β cDNA construct, which restores phosphorylation to endogenous acid hydrolases for transport to the lysosome [12], gave rise to the 48 kDa intermediate and 30 kDa mature heavy chain of cathepsin D (Fig. 3A, compare lanes 1 and 2). The WT parental HeLa cells in this experiment mainly contained the mature/heavy chain form of cathepsin D (Fig. 3A, lane 7). The lack of quantitative conversion of the pro‐cathepsin D to the mature 2‐chain form is most likely due to the low transfection efficiency of HeLa cells. Treatment of the transfected *GNPTAB* ^−/−^ cells with the selective cysteine protease inhibitor E‐64 completely abrogated cathepsin D processing at a concentration of 100 μm (Fig. 3A, lane 5). This concentration of E‐64 also mostly, but not completely, inhibited the formation of mature cathepsin D in the WT cells whereas 10 µm was without effect (Fig. 3A, compare lanes 9 and 10), in agreement with the previously published study using E‐64 to inhibit processing of cathepsin D [18]. This finding confirms the reports of others implicating cysteine proteases in the processing of cathepsin D [18, 19].

The finding that E‐64 prevented maturation of pro‐cathepsin D in the WT HeLa cells prompted us to ask whether the two cysteine proteases previously shown to mediate cathepsin D processing, namely cathepsin B and cathepsin L, are also present in *GNPTAB* ^−/−^ HeLa cells [18, 19]. Immunoblot analysis of whole‐cell lysates showed no detectable cathepsin B in the *GNPTAB* ^−/−^ cells whereas the WT and *GNPTG* ^−/−^ cells had readily detectable levels of this enzyme (Fig. 3B). It should be noted that while the parental HeLa cells show both the active 2‐chain form of cathepsin B (around 25 kDa) and the active single‐chain form (around 30 kDa) [20], only the latter form is detected in the *GNPTG* ^−/−^ cells. The reason for this discrepancy is not clear. The fact that *GNPTAB* ^−/−^ cells have the same level of *CTSB* mRNA as WT cells (Fig. 2A), but no detectable cathepsin B protein, suggests that all of the synthesized enzyme is likely being secreted in the absence of the M‐6‐P pathway. On the other hand, only pro‐cathepsin L was detected in all 3 cell lines with the anti‐cathepsin L antibody used in this study, even with the long exposure (Fig. 3B*).

### Cell uptake of cathepsin B facilitates processing of cathepsin D

We next asked whether delivery of exogenous cathepsin B to the lysosomes of the *GNPTAB* ^−/−^ cells could partially restore processing of the endogenous pro‐cathepsin D. To this end, cathepsins B, C, L, and Z were coexpressed with a truncated form of GlcNAc‐1‐phosphotransferase (S1‐S3) that exhibits high activity toward lysosomal enzymes in Expi293 cells [13]. These four cathepsins were selected on the basis of their expression in HeLa cells (Fig. 2A). We have previously shown that coexpression of a number of lysosomal enzymes with S1‐S3 in Expi293 cells resulted in the secretion of highly phosphorylated enzymes into the media [13]. Since the uptake of the secreted enzymes via the cell‐surface cation‐independent M‐6‐P receptor is dependent upon the extent of phosphorylation, we surmised that coexpression with S1‐S3 would result in the production of enzymes with a higher M‐6‐P content for use in the uptake experiments.

**Fig. 4:**
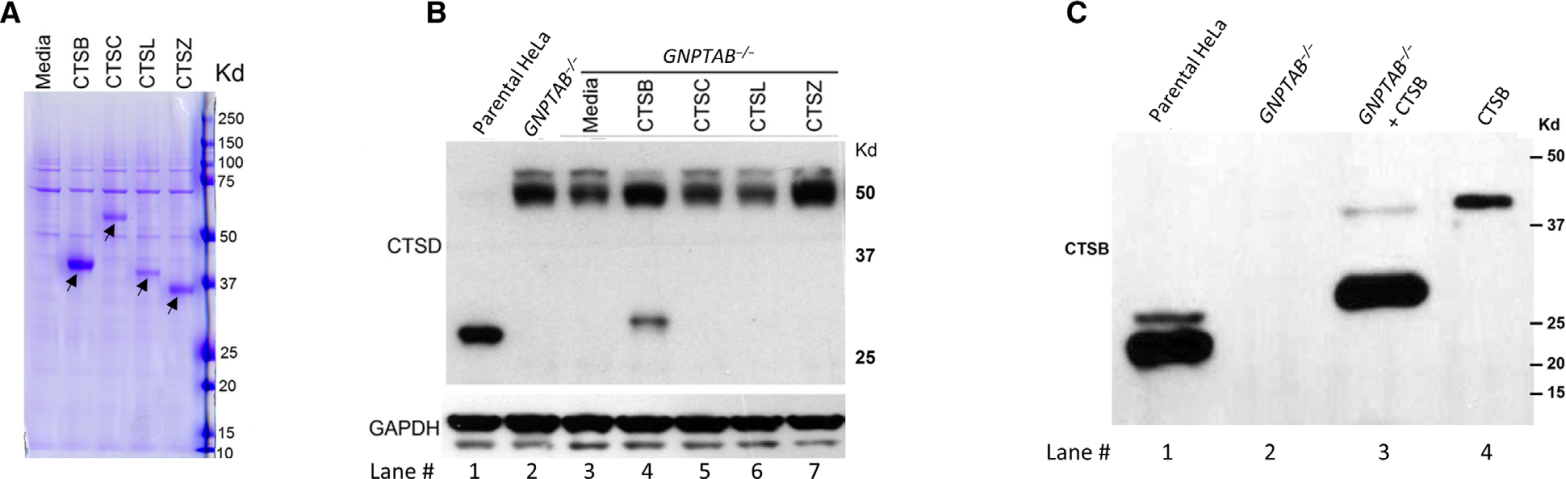
Cell uptake of exogenously added cathepsins. (A) Enzyme produced by Expi293 cells was collected from the media of cells transfected with the cDNAs of the respective cathepsins and cotransfected with the α/β precursor or the S1‐S3 mutant. Ten microlitre media were resolved by SDS/PAGE and the gel stained with Coomassie Brilliant Blue‐250. The position of the secreted cathepsins are indicated by black arrows. (B) Immunoblot of *GNPTAB* ^−/−^ cells following uptake of enzyme from the media, and probed for endogenous cathepsin D. (C) Immunoblot analysis of parental HeLa cells (lane 1), and untreated *GNPTAB* ^−/−^ cells (lane 2), or treated with media containing secreted cathepsin B (lane 3), and probed for cathepsin B. Ten microlitre of the Expi293 cell media containing the secreted cathepsin B was loaded in lane 4.

The media collected from these cultures contained high levels of each protease (Fig. 4A, arrows). The conditioned media were then added to cultures of *GNPTAB* ^−/−^ cells for 24 h. Immunoblot analysis of whole‐cell lysates probed for cathepsin D showed that only cathepsin B‐containing conditioned media facilitated processing of pro‐cathepsin D to the mature species (Fig. 4B, lane 4). Differential processing of the cathepsin D N‐linked glycans due to the lack of phosphorylation of the high mannose‐type sugar chains results in the slower migration of the cathepsin D heavy chain in *GNPTAB* ^−/−^ cells compared with WT HeLa cells (Fig. 4B, compare lanes 1 and 4) [21, 22].

The cathepsin B that was fed to the cells (Fig. 4C, lane 4) and taken up from the conditioned media was also processed to a mature species that migrated somewhat slower than the endogenous, mature cathepsin B in WT Hela cells (Fig. 4C, compare lanes 1 and 3). The basis for the discrepancy is not clear at this time.

## Discussion

Numerous studies published over the last several decades have implicated cathepsin D in a variety of physiological processes as well as diseased states [23]. Hence, a clear understanding of the gene regulatory networks underlying control of *CTSD* gene expression, as well as the mechanisms involved in the proteolytic processing of cathepsin D under normal and pathological conditions, is important. Here, we present evidence for the sustained elevation of *CTSD* gene expression at the transcriptional level when the M‐6‐P pathway is abrogated in HeLa cells. This lysosomal stress results in an approximately sixfold increase in the steady‐state message level of the *CTSD* gene that is still maintained when the transcription factors TFEB and TFE3 are simultaneously silenced by RNA‐i. In addition, GFP‐tagged TFEB or TFE3 did not show any discernible difference in subcellular localization between the WT parental and *GNPTAB* ^−/−^ HeLa cells (data not shown), in contrast to the enhanced nuclear translocation of TFEB that has been observed with renal glomerular cells isolated from *Gnptab* ^−/−^ mice [24]. Taken together, these results suggest that an alternate mechanism/s must be present in HeLa cells that contribute to the upregulation of gene expression due to inactivation of the α/β subunits of GlcNAc‐1‐phosphotransferase. The *GNPTAB* ^−/−^ HeLa cells have been subject to long‐term adaptation to lysosomal stress due to many passages of the cell line over time. The roles of TFEB and TFE3 in lysosomal regulation for cellular adaptation under a variety of acute stress conditions such as nutrient deprivation and pathogen infection are well documented [11]. However, this does not rule out the existence of additional pathways that may be involved in lysosomal homeostasis, especially when lysosome function is impaired in the long run.

Previously, we showed that the cathepsin D synthesized by *GNPTAB* ^−/−^ cells is not phosphorylated and is hyper‐secreted [12]. In view of the substantial increase in cathepsin D expression at both the mRNA and protein levels in these cells, it is possible that a fraction of the newly synthesized cathepsin D is nonspecifically incorporated into clathrin‐coated vesicles that bud off the TGN and are trafficked to the endosome/lysosome compartment. In addition, some of the cathepsin D may be targeted to the lysosome by a M‐6‐P‐independent mechanism [9, 25, 26]. Our finding that *GNPTAB* ^−/−^ cells are unable to process this retained pro‐cathepsin D to the mature heavy and light chains provided a useful system for searching for a protease that acts on this intermediate. The first clue that implicated cathepsin B came from immunoblot analysis of whole‐cell lysates which showed good expression of the cysteine protease cathepsin L, but no detectable cathepsin B, in the mutant cells. Since the mutant cells had similar levels of *CTSB* mRNA as WT cells, the lack of cathepsin B is presumably due to hypersecretion of the enzyme. Evidence for the role of cathepsin B in this process was then obtained by the cellular uptake experiment wherein conditioned media containing each of the five expressed cathepsins, which we show are synthesized by the *GNPTAB* ^−/−^ cells (Fig. 2A), were individually fed to this cell type. Our results show that only cathepsin B was capable of cleaving the endogenous pro‐cathepsin D. Consistent with a role for cathepsin B in this processing is the finding that the cysteine protease inhibitor, E‐64, blocked pro‐cathepsin D conversion in the WT HeLa cells, as well as in the *GNPTAB* ^−/−^ cells transfected with a cDNA construct encoding the α/β subunits of GlcNAc‐1‐phosphotransferase. Cathepsin L, in addition to cathepsin B, has been reported to be involved in pro‐cathepsin D maturation [18]. While our cell uptake data favor cathepsin B acting alone in this process, at least in HeLa cells, we cannot exclude a role for cathepsin L since our anti‐cathepsin L antibody detected only the proform of cathepsin L in both parental and *GNPTAB* ^−/−^ HeLa cells, and not the mature active form.

In conclusion, we present our data that inactivation of the M‐6‐P pathway in HeLa cells results in an upregulation of *CTSD* gene expression at the transcriptional level that occurs independent of TFEB and TFE3. In addition, we determine that it is the lack of cathepsin B in these *GNPTAB* ^−/−^ cells that is responsible for the failure of the retained cathepsin D to undergo proteolytic processing and maturation.

## Conflict of interest

The authors declare no conflict of interest.

## Author contributions

LL conceived and designed the project. LL acquired the data. LL and BD analyzed and interpreted the data. BD wrote the paper.

## Data Availability

Data will be available from the corresponding author upon reasonable request.
